# A Journey into the Clinical Relevance of Heme Oxygenase 1 for Human Inflammatory Disease and Viral Clearance: Why Does It Matter on the COVID-19 Scene?

**DOI:** 10.3390/antiox11020276

**Published:** 2022-01-29

**Authors:** Ayelen Toro, María Sol Ruiz, Sofia Lage-Vickers, Pablo Sanchis, Agustina Sabater, Gaston Pascual, Rocio Seniuk, Florencia Cascardo, Sabrina Ledesma-Bazan, Felipe Vilicich, Elba Vazquez, Geraldine Gueron

**Affiliations:** 1 Laboratorio de Inflamación y Cáncer, Departamento de Química Biológica, Facultad de Ciencias Exactas y Naturales, Universidad de Buenos Aires, Buenos Aires C1428EGA, Argentina; ma.sol.ruiz@qb.fcen.uba.ar (M.S.R.); sofilage@qb.fcen.uba.ar (S.L.-V.); pablosanchis@qb.fcen.uba.ar (P.S.); asabater@qb.fcen.uba.ar (A.S.); gpascual@qb.fcen.uba.ar (G.P.); rseniuk@qb.fcen.uba.ar (R.S.); fcascardo@qb.fcen.uba.ar (F.C.); sledesmabazan@qb.fcen.uba.ar (S.L.-B.); fvilicich@qb.fcen.uba.ar (F.V.); elba@qb.fcen.uba.ar (E.V.); 2 CONICET-Universidad de Buenos Aires, Instituto de Química Biológica de la Facultad de Ciencias Exactas y Naturales (IQUIBICEN), Buenos Aires C1428EGA, Argentina

**Keywords:** heme oxygenase 1, COVID-19, influenza A virus, respiratory syncytial virus, human immunodeficiency virus, Ebola virus, Dengue virus, Zika virus, Hepatitis virus, SARS-CoV-2

## Abstract

Heme oxygenase 1 (HO-1), the rate-limiting enzyme in heme degradation, is involved in the maintenance of cellular homeostasis, exerting a cytoprotective role by its antioxidative and anti-inflammatory functions. HO-1 and its end products, biliverdin, carbon monoxide and free iron (Fe^2+^), confer cytoprotection against inflammatory and oxidative injury. Additionally, HO-1 exerts antiviral properties against a diverse range of viral infections by interfering with replication or activating the interferon (IFN) pathway. Severe cases of coronavirus disease 2019 (COVID-19), an infectious disease caused by severe acute respiratory syndrome coronavirus 2 (SARS-CoV-2), are characterized by systemic hyperinflammation, which, in some cases, leads to severe or fatal symptoms as a consequence of respiratory failure, lung and heart damage, kidney failure, and nervous system complications. This review summarizes the current research on the protective role of HO-1 in inflammatory diseases and against a wide range of viral infections, positioning HO-1 as an attractive target to ameliorate clinical manifestations during COVID-19.

## 1. Introduction

Severe acute respiratory syndrome coronavirus 2 (SARS-CoV-2) emerged in late 2019 in Wuhan, China. The World Health Organization (WHO) declared coronavirus disease 2019 (COVID-19) a pandemic health emergency as of 31 January 2020. The treatment goal in COVID-19 patients is to prevent or to decrease the strong virus induced inflammatory stimuli associated with a wide spectrum of poor prognosis clinical manifestations [1]. Heme oxygenase 1 (HO-1) is a microsomal enzyme with a primary antioxidant and anti-inflammatory role involved in heme degradation, generating carbon monoxide (CO), biliverdin (BV), and free iron (Fe^2+^) [2]. Hence, HO-1 induction is a useful approach for inflammatory diseases treatment [3,4,5,6]. Additionally, HO-1 displays antiviral properties against a wide range of viruses [7]. Hemin, a previously Food and Drug Administration (FDA) and European Medicines Agency (EMA) approved drug for acute intermittent porphyria treatment [8,9], is a well known inducer of HO-1 that increases its plasma concentration in humans. Thus, hemin rises as a promising drug candidate against the replication of different viruses, including SARS-CoV-2. In this review, we summarize the current research on the protective role of HO-1 in inflammatory diseases and in a diverse range of viral infections, positioning this protein as a potential therapeutic target to ameliorate COVID-19′s clinical manifestations.

## 2. Re-Emergence of the Coronavirus Disease

Coronaviruses (CoVs) are a large family of positive sense, single stranded RNA (+ssRNA) viruses that infect humans, other mammals, and birds, causing respiratory, enteric, hepatic, and neurologic diseases [10,11]. CoVs first became renowned in 2002–2003 during an outbreak of a virus with zoonotic origin; the severe acute respiratory syndrome coronavirus (SARS-CoV) originated in China, with 8096 cases and 774 deaths reported between 2002 and 2003 [12], and a case–fatality ratio of 7.2% [13]. In 2012, the Middle East respiratory syndrome coronavirus (MERS-CoV), another virus with zoonotic origin, emerged in Saudi Arabia and caused 927 fatalities among 2581 registered cases [14,15].

By the end of 2019, the Wuhan Health Commission from China reported a number of pneumonia cases of unknown cause and varying severity in the city of Wuhan, China. High throughput sequencing allowed the quick identification of a novel CoV belonging to the beta-coronavirus family, which was named severe acute respiratory syndrome coronavirus 2 (SARS-CoV-2), as the causative agent of Coronavirus disease 2019 (COVID-19) [16]. This pathogen rapidly spread globally via travel related cases; constituting a pandemic and setting an immense challenge for public health [16]. Unlike SARS-CoV and MERS-CoV, SARS-CoV-2 can be transmitted among people before the onset of symptoms or from asymptomatic individuals [17], limiting effective control of the spread. As of 30 December 2021, the WHO reports 281,808,270 confirmed cases and 5,411,759 deaths worldwide [15].

Although COVID-19 is primarily a respiratory disease, SARS-CoV-2 has the capacity to infect a broad range of cell types in different organs and systems, including the central nervous system [18]. SARS-CoV-2 infection begins in the proximal airways and could trigger severe and sometimes fatal symptoms when reaching the distal lung [19]. Among the severe respiratory diseases caused by SARS-CoV-2, acute respiratory distress syndrome (ARDS) and acute lung injury (ALI) [20,21] are complications mainly caused by an exacerbated immune response in elders and patients with comorbidities [22]. Cytokine storm contributes to ALI and the development of ARDS in patients with severe pneumonia caused by SARS-CoV-2, as well as SARS-CoV and MERS-CoV [23,24]. Surprisingly, in addition to the respiratory symptoms, patients may suffer from cardiac, hematological, neurological, hepatic, gastrointestinal and kidney complications [25]. COVID-19 may result in long term sequelae characterized by organ injuries that cannot be completely reversed. Several patients, even those with mild cases, may develop lasting symptoms that can have disabling consequences [26,27].

The COVID-19 era is far from being constrained, and the emergence of new viral variants causing future outbreaks remains a threat. As of December 2021, the WHO has defined five variants of concern (Alpha (B.1.1.7), Beta (B.1.351), Gamma (P.1), Delta (B.1.617.2), and Omicron (B.1.1.529)), as well as two variants of interest (Lambda (C.37) and Mu (B.1.621)) [15]. It has been reported that some variants, such as the recently described Omicron [28], have higher transmission rates and are able to escape neutralizing antibodies generated by natural infection and vaccination, or therapeutic antibodies [29,30,31,32]. Incomplete knowledge on the pathogenesis of SARS-CoV-2 and the diverse array of symptoms and manifestations of COVID-19 pose a great challenge for the development of effective treatments that should mainly focus on both, decreasing viral replication and modulating the immune response.

## 3. Cytokine Storm and Inflammation

Inflammation involves defense mechanisms against infection or injury. It is responsible for activating both innate and adaptive immune responses [33,34]. During infections, innate cells recognize pathogen associated molecular patterns (PAMPs) from the invading agent. In the case of inflammation triggered by tissue damage, trauma or ischemia, innate cells recognize host specific molecules that are released during cell injury or necrotic death, defined as damage associated molecular patterns, such as nucleic acids and adenosine triphosphate (ATP) [33]. During the early stages of inflammation, innate immune cells and endothelial cells (EC) release a diverse set of cytokines: chemotactic cytokines, such as monocyte chemotactic protein-3 (MCP-3) and interferon (IFN) γ-induced protein 10 (IP-10), and recruit other immune cells to the site of infection or inflammation. Proinflammatory cytokines, such as tumor necrosis factor alpha (TNF-α), interleukin-6 (IL-6), and IL-1β [35,36], are also released and trigger the activation of inflammatory pathways, including the mitogen activated protein kinase (MAPK), nuclear factor kappa-B (NF-κB), and Janus kinase (JAK)-signal transducer and activator of transcription (STAT) pathways [34]. Some pathogenic viruses (i.e., highly virulent subtypes of influenza) and bacteria (i.e., *Francisella tularensis*) can induce the acute dysregulated production of inflammatory cytokines, known as “cytokine storm” or hypercytokinemia [37]. The hypercytokinemia and exacerbated secondary events, such as coagulation, eventually result in widespread necrosis, organ failure and death [33,38].

Once SARS-CoV-2 infects target cells, innate immune cells are recruited to the infection site, where they release cytokines and initiate the activation cascade of adaptive B and T cell immune responses [39]. In most cases, the immune system is able to eliminate virus infected cells and resolve the immune response. However, in some patients, this process is dysfunctional, impairing the effective clearance of infected cells, and causing severe damage to the host [40].

## 4. The Lead Role of Interferons upon Viral Infection

During viral infections, pattern recognition receptors are stimulated to produce IFN by the innate immune cells. IFNs are crucial for the induction of an antiviral state via autocrine and paracrine signaling. There are three types of IFNs: type I, type II and type III. Type I (IFNα, IFNβ, IFNω, IFNτ, IFNε) and Type III (IFNλ1, IFNλ2/3, IFNλ4) share similar dynamics after binding to its receptor, as cross-phosphorylation between JAK1 and tyrosine kinase 2 (TYK2) occurs [41]. Subsequently, a docking site for STAT1 is exposed, STAT1 is phosphorylated, translocates to the nucleus, and induces the transcription of interferon stimulated genes (ISGs). The IFNs biological effects are wide, including immuno-regulation, antiviral, anti-angiogenic, and pro-apoptotic functions [42]. However, many pathogens have evolved to elude the action mechanisms of these powerful cytokines [43,44,45].

In critically ill COVID-19 patients, a hyperinflammation state prevails. In March 2020, a retrospective study on 150 patients from Wuhan, China, found elevated levels of IL-6 and C-reactive protein (CRP) in SARS-CoV-2 infected patients that died compared with discharged patients [46]. An independent report based on 50 COVID-19 patients with moderate to severe disease, identified IP-10, MCP-3, and IL-1 receptor antagonist (IL-1ra) as independent predictors for disease severity [47]. A longitudinal analysis showcased IL-18 and IFN-α as top biomarkers for predicting mortality. Consequently, higher counts of inflammatory monocytes, plasmablast like neutrophils and eosinophils have been described in patients with severe disease [39].

Blanco-Melo et al. reported an impairment in the response of type I and type III IFNs against SARS-CoV-2 infection [40]. In contrast, a recent study found that severe COVID-19 cases showed an exacerbated expression of type I IFNs, which could lead to augmented inflammation [48]. Several clinical trials evaluating IFNs have been carried out in COVID-19 patients. Two different studies showed a reduction in the mortality rate after IFNβ-1a and IFNβ-1b treatment [49,50]. Another study, using IFNα-2b, reported a decrease in detectable SARS-CoV-2 in the upper respiratory tract associated with lower inflammatory cytokines levels, such as IL-6 and CRP [51]. In addition, peginterferon λ treatment was associated with a reduction in viral RNA [52]. Furthermore, there are several ongoing clinical trials, using recombinant human IFNs or IFNs combined with other drugs [53,54,55]. These evidences highlight IFNs as potential targets for COVID-19 treatment. In the next section we will focus on the stimulation of IFN pathway by HO-1 induction.

## 5. Understanding the Protective Role of Heme Oxygenase 1

Heme oxygenases (HO) are metabolic enzymes that partake in the degradation of the heme group [2]. To date, three isoforms of this protein have been found: HO-1, which can be induced by external factors (such as hypoxia, oxidative stress, heat shock, reactive oxygen species (ROS), among others) [56]; HO-2, a constitutively expressed isoform; and HO-3, a nonfunctional isoform in humans [57].

In particular, HO-1, encoded by the *HMOX1* gene, is involved in the maintenance of cellular homeostasis, exerting a cytoprotective role by its anti-inflammatory, anti-oxidative and anti-apoptotic functions, as revealed in a human case of genetic HO-1 deficiency [58]. This enzyme participates not only in normal physiological processes, but also performs a protective role in inflammatory physiopathological conditions, such as kidney disease [59], cancer [60,61], cardiovascular disease [62], asthma [63] and inflammatory bowel diseases [4,64].

HO-1 is expressed in most cell types and tissues; however, its capacity to counteract inflammation seems to be critically dependent on its specific functions in myeloid cells and in EC [65]. In myeloid cells, HO-1 acts as a key regulator of the TLR4/TLR3/IRF3 induced production of IFN-β and primary IRF3 target genes in macrophages [66] and modulates maturation and specific functions of dendritic cells [67,68]. Moreover, HO-1 over-expression in macrophages negatively regulates the expression of diverse proinflammatory molecules and increases the expression of anti-inflammatory cytokines [69,70,71]. Among HO-1 effects on EC, it is significant to mention its ability to inhibit the expression of pro-inflammatory genes related to EC activation, such as the TNF-α-induced adhesion molecules, E-selectin and VCAM-1, via a mechanism associated with the inhibition of NF-κB activation [72].

HO-1 cleaves the heme group generating BV, CO and Fe^2+^. Heme is usually bound to a myriad of proteins and it is involved in several homeostatic functions [56]. However, elevated concentrations of heme can cause cell damage because it is a pro-oxidant molecule. It can diffuse through cell membranes and deliver a redox active iron, producing ROS [73]. Excessive amounts of these molecules are toxic and induce oxidative stress that, in turn, generates DNA and protein damage, aggregation and lipid peroxidation, triggering cells permeability and driving cell lysis and death [73].

Several studies highlight heme catabolism end products as potential therapeutic targets in vascular disease, based on their anti-inflammatory and antiproliferative functions [74]. BV and its reduced form, bilirubin (BR), are powerful antioxidants that are able to scavenge ROS and counteract the oxidative stress. BV and BR are critical for the regulation of inflammation by exerting immunosuppressive effects [75], as they have been reported to have potent anti-inflammatory activity against insulin resistance by reducing visceral obesity and adipose tissue inflammation [76].

In addition, CO is considered an anti-apoptotic [77], antiproliferative and anti-inflammatory factor [78]. CO contributes to blood vessel development [79] and promotes angiogenesis, a crucial process involved in tissue reparation after a pathological state [80]. Interestingly, CO also reduces inflammation and inhibits apoptosis by interacting with antigen-presenting cells and suppressing T cell proliferation [81]. Moreover, it has been reported that it downregulates proinflammatory cytokines via the p38/MAPK pathway in RAW 264.7 macrophages and C57BL/6 mice [70], by the c-Jun N-terminal kinase (JNK) pathway in a murine model of sepsis [82] and through the extracellular signal regulated kinase (ERK) signaling pathway in CD4^+^ T cells [81]. Further, HO-1/CO induced downregulation of the NLRP3 (NOD-, LRR- and pyrin domain-containing protein 3) inflammasome activation has been demonstrated in different models of murine hepatic and lung inflammatory injury [83,84,85]. 

Moreover, the HO-1 mediated increase in Fe^2+^ concentration upregulates the expression of ferritin, an iron chelating protein [86]. Ferritin exerts antioxidative and cytoprotective effects [74], as this product scavenges redox active Fe^2+^, rendering it not harmful for cells and avoiding subsequent production of ROS via Fenton reaction. Fe^2+^ performs its function by inhibiting IL-2 and IgG production, and downregulating the MAPK and NF-κB signaling pathways [56,75,87].

### Therapeutic Potential of HO-1 Induction to Treat Chronic Inflammation

**Figure 1:**
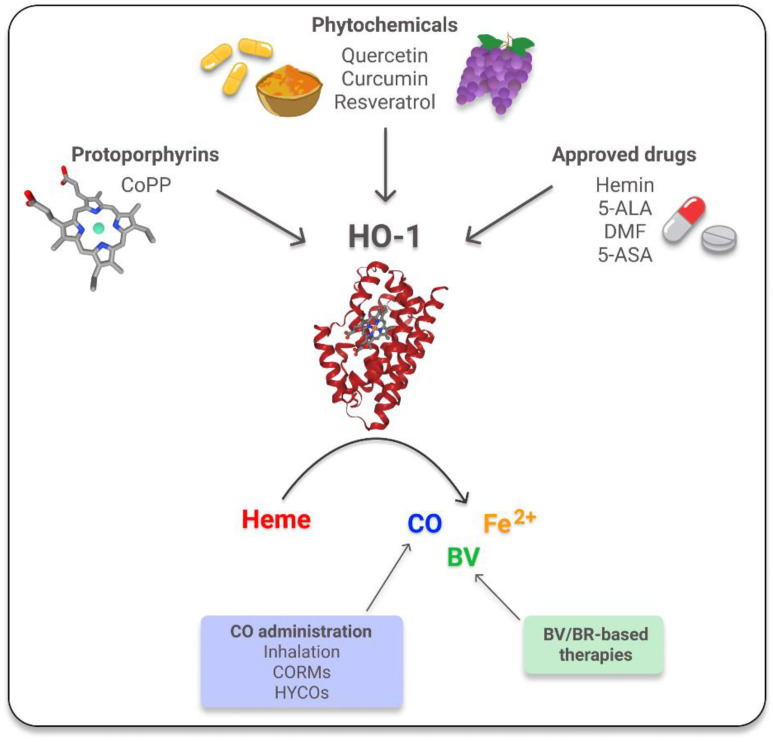
Inducers of HO-1. HO-1 degrades heme producing equimolar amounts of carbon monoxide (CO), biliverdin (BV) and Fe^2+^. HO-1′s inducers are grouped into protoporphyrins, a type of porphyrins that forms heme; phytochemicals, natural antioxidants compounds contained in plants; and approved drugs, compounds that were previously approved by the FDA. CoPP: cobalt protoporphyrin IX; 5-ALA: 5-aminolevulinic acid; DMF: dimethyl fumarate; 5-ASA: 5-aminosalicylic acid; CORMs: CO-releasing molecules; HYCOs: Hybrid carbon monoxide-releasing molecules; BR: bilirubin. The images of HO-1 and CoPP were taken from RCSB PDB (PDB ID: 1N3U) and The National Center for Biotechnology Information [107,108].

As HO-1 and its reaction products exert protective anti-inflammatory effects in different preclinical models [3,4,5,88,89,90], the induction of the HO-1 system has emerged as a promising potential therapy for chronic inflammatory diseases. Most of the studied strategies are based on the use of the traditional pharmacological inducers: hemin [91,92], an FDA and EMA approved drug, and cobalt protoporphyrin IX (CoPP) [5,93,94]. In addition, many phytochemicals, such as quercetin, curcumin and resveratrol, are currently under investigation, due to their potential as HO-1 alternative inducers to counteract inflammation processes with lower cytotoxic secondary effects [95,96,97]. Alternatively, there are also a few approved drugs, such as 5-aminosalicylic acid (5-ASA), dimethyl fumarate (DMF), and 5-aminolevulinic acid (5-ALA), whose beneficial properties in inflammatory conditions are explained, at least in part, by their capacity to induce HO-1 [96,98,99] (Figure 1). Additionally, another effective option is the use of BV/BR based therapies, which have proven to be effective for these chronic pathologies [100,101,102] and/or the direct administration of CO via inhalation, CO-releasing molecules (CORMs) or hybrid carbon monoxide-releasing molecules (HYCOs). HYCOs are a type of compound where CORMs are combined with DMF, causing a powerful anti-inflammatory action due to its effect on the NRF2/HO-1 pathway [5,100,103,104,105,106] (Figure 1).

## 6. HO-1 Mechanism of Action against Inflammatory Lung Diseases

**Figure 2:**
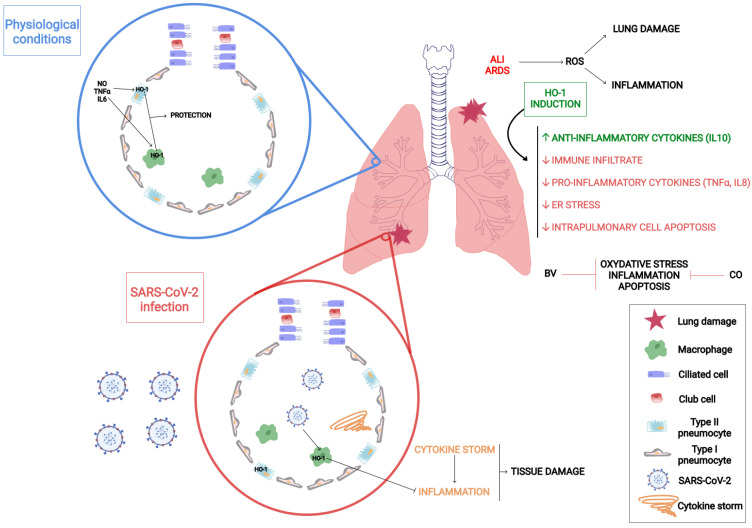
HO-1 and inflammatory lung diseases. HO-1 is expressed in pulmonary cells and confers protection against inflammatory lung diseases such as acute respiratory distress syndrome (ARDS), acute lung injury (ALI), and SARS-CoV-2 infection. Schematic representation displaying HO-1′s reaction and its products’ protective effects in the lung tissue.

There is extensive literature about the role of HO-1 in lung diseases. This protein is expressed in type II pneumocytes and in alveolar macrophages and contributes to the protection of the lung tissue. The main HO-1 inducers in the lungs are pro-inflammatory cytokines, such as TNF-α and IL-6, the heme group and nitric oxide (NO), as well as hypoxia and hyperoxia conditions [109] (Figure 2). There is sound evidence that states that HO-1 induction is a critical defense factor during acute and chronic lung processes [109,110,111].

As mentioned earlier, during COVID-19 disease, the number of immune cells infiltrating lung tissues and the pro-inflammatory cytokines levels are augmented [112]. Consequently, anti-inflammatory proteins have a crucial role in halting the cytokine storm and the sequelae generated by viral infection [113].

In particular, ALI and ARDS are the most prevalent diseases emerging from an extended diversity of lung injuries [114,115]. ALI and ARDS are pathophysiologically characterized by lung damage, inflammatory infiltration, and an exacerbation of the host immune response [116]. Several reports indicate that ALI and ARDS might be explained by the presence of high ROS levels, where HO-1 acts as a protective factor against oxidative stress under pharmacological induction [117]. HO-1 induction by hemin shows a protective role against ventilator induced lung injury in rabbits with ALI/ARDS, increasing anti-inflammatory cytokine levels, such as IL-10, as well as decreasing the inflammatory infiltrate of immune system cells and the secretion of inflammatory cytokines, such as TNF-α and IL-8 [118] (Figure 2). Furthermore, it has been found that HO-1 confers protection against ischemia-reperfusion injury (LIRI) [119].

HO-1 regulates diverse signaling pathways that are affected during pulmonary diseases. In rats, HO-1 inhibits the PERK/eIF2-α/ATF4/CHOP pathway, which is involved in the endoplasmic reticulum stress (ERS) characteristic in ALI, and also promotes the decrease in intrapulmonary cell apoptosis [120]. It was also reported that the PI3K/Akt pathway attenuates oxidative damage during ALI/ARDS through HO-1 regulation [121]. In pathologies such as silicosis, characterized by excessive ROS production, lung injury is attenuated by HO-1 induction. The mechanism underlying this cytoprotective effect relies on the ERK pathway inhibition by HO-1, CO and BV [122].

Reaction products derived from the HO-1 mediated heme catalysis have protective roles in lung pathologies as well. CO is known to provide protection against ALI and ARDS by reducing cytokine and chemokine levels [105,117,123]. CO decreases EGR-1 (early growth response protein 1), a proinflammatory protein that regulates the expression of TNF-α and IL-2 [124], in mice lungs [123]. Fujita et al. demonstrated that *Hmox1* deficient mice had increased mortality after lung ischemia, an effect reverted by CO administration [125]. Furthermore, BV exerted antioxidative, anti-inflammatory and anti-apoptotic effects in a rat model of LIRI [126]. BV administration protected against hemorrhagic shock induced ALI through a decrease in the inflammatory infiltrate and proinflammatory cytokines levels [127].

## 7. Unveiling How HO-1 Promotes Viral Clearance

**Figure 3:**
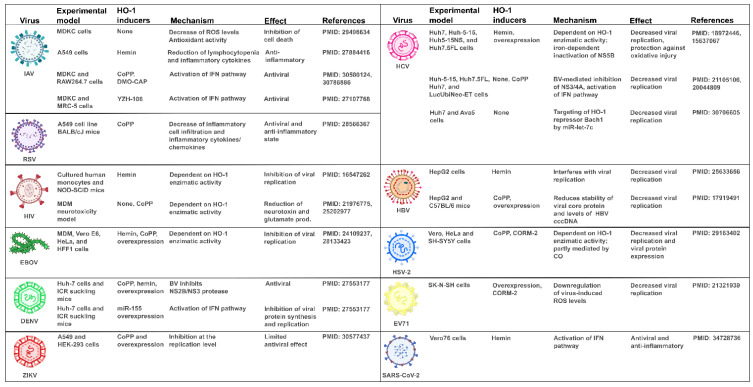
HO-1′s induction and its effect on different viral infections. Table containing previously reported studies about HO-1 involvement in influenza A virus (IAV), respiratory syncytial virus (RSV), human immunodeficiency virus (HIV), ebola virus (EBOV), dengue virus (DENV), zika virus (ZIKV), hepatitis C virus (HCV), hepatitis B virus (HBV), herpes simplex virus 2 (HSV-2), enterovirus 71 (EV71) and severe acute respiratory syndrome coronavirus 2 (SARS-CoV-2) infections. The table includes the experimental model, HO-1 inducers, its mechanism of action, its effect and the study’s PMID. CoPP: cobalt protoporphyrin IX, DMO-CAP: 6-demethoxy-4′-O-methylcapillarisin, ROS: Reactive oxygen species, IFN: interferon, MDM: monocyte derived macrophages, BV: Biliverdin, CO: carbon monoxide, CORM-2: CO-releasing molecule-2.

HO-1 has immunomodulatory properties on the innate immune response and there is compelling evidence suggesting that it also plays a central role in the modulation of adaptive immunity. HO-1 displays antiviral properties against a wide range of viruses [7] (Figure 3). Several reports have demonstrated that HO-1 induction is associated with the activation of the IFN pathway. However, the mechanism underlying the antiviral properties of HO-1 exerted by both its classical and noncanonical activities are yet to be fully elucidated.

### 7.1. Respiratory Viruses: IAV and RSV

Influenza A virus (IAV) is a single stranded RNA virus whose infection remains a persistent global health threat with high morbidity and mortality [128]. An estimate of 4.0 to 8.8 deaths per 100,000 individuals with seasonal influenza associated respiratory occur annually (all types of influenza virus) [129]. Considering that the inhibition of virus induced ROS formation impairs IAV replication, proteins such as HO-1 are useful to counteract IAV infections in the host cell [130]. Wang et al. studied the effect of hemin in IAV infections and demonstrated that hemin attenuates the lymphocytopenia caused by IAV infection both in vitro and in vivo [131]. These results suggest that the anti-influenza effect of hemin may be mediated by HO-1′s ability to regulate systemic and local inflammatory responses [131]. Furthermore, Hashiba et al. reported that HO-1 gene transfer is a potential strategy to treat lung injury caused by IAV [132] and Cummins et al. suggested that the therapeutic induction of HO-1 expression may represent a novel adjuvant to enhance influenza vaccine effectiveness [133]. It has also been reported that the HO-1 inducers rupestonic acid derivative YZH-106 and the flavonoid 6-demethoxy-4′-O-methylcapillarisin (DMO-CAP) inhibited IAV replication by activating the HO-1 mediated IFN response [134,135]. Additionally, Ma et al. evaluated the effect of CoPP in IAV infection, focusing on the IFN pathways. The authors demonstrated that HO-1 induction attenuates IAV replication, and the most intriguing finding was that the catalytic function of HO-1 was not essential for the anti-IAV effect of CoPP. Interestingly, they found that HO-1 interacts with IFN regulatory factor 3 (IRF3) promoting its phosphorylation and nuclear translocation, thus activating the IFN pathway. Consequently, CoPP treatment increased the expression of *IFITM3*, *PKR* and *OAS1*, three ISGs markedly involved in the anti-IAV response [128].

Respiratory syncytial virus (RSV) is an RNA virus of the Pneumoviridae family and the most common cause of lower respiratory tract infections in children worldwide [136]. It interacts with host cells’ toll-like receptors in the primary airway epithelium, and promotes the expression and secretion of inflammatory cytokines [137], under the NF-κB pathway’s regulation [138]. Similar to IAV, CoPP HO-1 induction inhibited RSV replication and viral particle production in lung adenocarcinoma (A549) and HEp-2 cells. Most importantly, in vivo assays in BALB/cJ mice treated prophylactically with CoPP also showed a reduction in viral replication and viral particle production, alongside a decrease in inflammatory cell infiltration, and inhibition of proinflammatory cytokine or chemokine secretion during RSV infection [139].

The mentioned reports suggest that HO-1 is involved in host cellular defense mechanisms against IAV and RSV infections. Of note, HO-1’s antiviral effects are mediated by its classical and noncanonical functions.

### 7.2. Retroviridae: HIV

The human immunodeficiency virus-1 (HIV-1) genome consists of two identical single stranded RNA molecules and it is the causative agent of acquired immune deficiency syndrome (AIDS). HIV infection has become a clinically manageable disease since the development of combination antiretroviral therapy (ART). Globally, 1 million people died from HIV/AIDS in 2016; without ART, more than twice as many people would have died from this disease [140]. In 2020, the United Nations Programme on HIV/AIDS (UNAIDS) reported that of all people with HIV worldwide, 66% were virally suppressed [141,142]. However, emerging drug resistance and limitations in access and adherence to ART impose a threat to controlling the spread of the virus [143]. Current drugs do not eradicate the virus, making lifelong treatment necessary [143].

Several reports from in vitro and in vivo models, and HIV infected subjects, have linked HO-1 with HIV replication and its effects on HIV mediated neurodegeneration. In cultured monocytes and HIV infected mice, hemin efficiently inhibited viral replication; the effect observed in vitro was mediated by HO-1 enzymatic activity [144]. Using an in vitro model of HIV mediated neurotoxicity, HIV infection dysregulated the macrophage antioxidant response and reduced levels of HO-1. Restoration of HO-1 expression in HIV-infected monocyte derived macrophages (MDM) reduced neurotoxin release without altering HIV replication [145]. In HIV infected subjects, HO-1 protein levels were reduced in the dorsolateral prefrontal cortex, which correlated with central nervous system (CNS) viral load markers of immune activation. In a model of human astrocytes treated with IFNγ, an HIV associated CNS immune activator, HO-1 was degraded by the immunoproteasome [146]. Additionally, the use of CoPP reduced HIV-MDM glutamate release and neurotoxicity, suggesting a role for HO-1 in HIV associated neurocognitive disorders pathogenesis [147].

Altogether, these reports propose HO-1 induction as a protective mechanism against HIV infection. In particular, the HO-1′s classical functions mediate its antiviral properties against HIV.

### 7.3. Filoviridae: EBOV

Ebola virus (EBOV) is a negative-sense RNA virus that belongs to the Filoviridae family [148,149]. From 2013 to 2015 there was an important outbreak in West Africa, which caused >25,000 infections and >10,000 deaths. The average EBOV case fatality rate is ~50% and case fatality rates has ranged from 25% to 90% in past outbreaks [15]. Hemin treatment significantly reduced EBOV replication and delayed pathogenesis in vitro, by stimulating the cellular innate response against the infection [150,151]. In MDM, hemin treatment inhibited EBOV infection in a dose dependent manner. A similar effect was observed in other cell lines, such as HeLa and human foreskin fibroblasts cells, in which hemin also reduced viral replication [150]. Furthermore, it has been reported that the ebola virus protein 35 (VP35) is a critical protein involved in the inhibition of IRF3 phosphorylation as a mechanism that might counteract the antiviral response [152]. Thus, considering that HO-1 promotes IRF3 phosphorylation and activation, its induction may represent a novel therapeutic strategy against EBOV infection.

The cited studies place HO-1 as a novel therapeutic target against EVOB infection. Notably, HO-1′s noncanonical functions are involved in the present example of antiviral action.

### 7.4. Hepatic Viruses: HCV and HBV

Hepatitis C virus (HCV), a single stranded positive sense RNA virus, is associated with chronic hepatitis, cirrhosis, steatosis and hepatocellular carcinoma [153]. HCV treatment includes a combination of pegylated IFN-α and ribavirin, which has low efficacy and important side effects. With the development of direct-acting antiviral agents, such as sofosbuvir and simeprevir, patient outcomes have greatly improved; however, the disease remains a concern [154]. Since HCV infection generates oxidative damage to hepatocytes, the modulation of HO-1 expression emerges as an attractive therapeutic approach to reduce chronic liver disease. Abdalla et al. observed lower HO-1 mRNA and protein levels in HCV infected patients’ livers, while this alteration was not found in patients with other chronic liver diseases. The authors also reported HO-1 downregulation in hepatocyte cell lines expressing the HCV core protein [155]. Further, the overexpression or hemin-induction of HO-1 in the hepatoma cell line Huh7 decreased HCV replication and conferred protection against oxidative injury [156]. This effect of HO-1 on HCV replication might be explained partly by the iron dependent inactivation of the HCV RNA polymerase NS5B [157], and by the BV mediated inhibition of HCV NS3/4A protease and induction of an antiviral response by IFNα2 and IFNα17 [153,158]. Moreover, overexpression of miR-let-7c, which interferes with the production of proinflammatory cytokines in osteoarthritis and rheumatoid arthritis synovial fibroblasts [159], can reduce HCV replication by targeting HO-1 transcriptional repressor Bach1 [160].

Hepatitis B virus (HBV) is a DNA virus that causes serious liver diseases, representing the most common etiological agent for these pathologies [161]. It has been shown that pharmacological and genetic HO-1 overexpression attenuates viral replication both in vivo and in vitro in HepG2 cells [161,162,163], while also playing a hepatoprotective role [162]. The effect of HO-1 induction using hemin and CoPP mitigated the effects of HBV replication [6,161,162]. On the other hand, blocking HO-1 by siRNA reversed the inhibition of viral replication [6]. Interestingly, Protzer et al. evaluated the effect of HO-1 on HBV core protein by pulse-chase metabolic labeling experiments finding that HO-1 can destabilize structural proteins to prevent the formation of viral capsids, highlighting a direct HO-1 antiviral mechanism rather than limiting its effect to its anti-inflammatory properties [6].

Hence, the summarized reports established the antiviral effects of HO-1 by impairing HCV’s and HBV’s replication.

### 7.5. Arbovirus: DENV and ZIKV

Dengue virus (DENV) is a single stranded positive sense RNA virus [164] that induces oxidative stress by the activation of inflammatory regulators, such as NF-κB, and leads to the progression and pathogenesis of DENV [165]. In this pro-oxidant scenario, Tseng et al. demonstrated that HO-1 promoter activity and protein synthesis gradually increased during the early stages of DENV infection (6 to 12 h), but they were markedly decreased at later stages (24 to 72 h) [166]. Strikingly, pharmacological and genetic HO-1 induction after infection impaired viral protein synthesis and replication, and reduced DENV mortality. This effect was due to BV but not CO nor Fe^2+^ production [166]. The authors demonstrated that BV inhibits NS2B/NS3 DENV protease, thus promoting the antiviral IFN response and impairing its blockage by this protease [166]. Accordingly, Su et al. showed the anti-DENV activity of miR-155, which inhibits Bach1, a protein that negatively regulates the expression of many oxidative stress-response genes, including *HMOX1* [167]. This, in turn, results in the induction of HO-1, boosting the IFN responses against DENV replication by the activation of interferon induced protein kinase R (PKR), 2’-5’-oligoadenylate synthetase 1 (OAS1), OAS2, and OAS3 expression [167]. Interestingly, the summarized studies demonstrate that HO-1′s antiviral effects against DENV infection involve both, its canonical, in this case mediated by BV, and noncanonical functions.

Zika virus (ZIKV), a single stranded positive sense RNA, is the causative pathogen of Zika fever in humans [168]. Using A549 and embryonic kidney (HEK-293) cell lines, El Kalamouni et al. demonstrated that ZIKV infection downregulated HO-1 expression by triggering endoplasmic-reticulum-associated protein degradation, thus halting its antiviral effects [168]. This report highlights HO-1′s protective role relevance, as it demonstrates that ZIKV infection promotes the decrease in HO-1 expression levels as an evasion mechanism.

### 7.6. Neurotropic Viruses: HSV-2 and EV71

Herpes simplex virus (HSV) includes HSV-1 and HSV-2, two double stranded DNA viruses that belong to Herpesviridae family. HSV produces recurring lesions in skin and mucosae and can also latently infect neurons of the trigeminal or dorsal root ganglia. HSV infection can result in encephalitis and meningitis [169]. Ibañez et al. demonstrated that pharmacological induction of HO-1 by CoPP hampered HSV-2 propagation in epithelial and neuronal cells. Furthermore, by CORM-2 treatment the authors also showed that the effects of HO-1 were partly mediated by CO [170].

Enterovirus 71 (EV71) is a single stranded positive sense RNA virus that belongs to the Picornaviridae family and is the causative agent of hand foot and mouth disease in children [171]. It was demonstrated that the overexpression of HO-1, as well as CO treatment, decreased viral replication in SK-N-SH cells suggesting that the antiviral effect is mediated by the downregulation of EV71 induced ROS levels [172].

Regarding neurotropic viruses, the summarized reports showcase that HO-1 displays protective effects against HSV-2 and EV71 involving its enzymatic function.

### 7.7. COVID-19 Causative Agent: SARS-CoV-2

SARS-CoV-2, is the novel beta coronavirus of the Coronaviridae family whose genome is composed of a single stranded RNA molecule [173]. It has been shown that hemin, hemoglobin and protoporphyrin IX bind to SARS-CoV-2 proteins, blocking its adsorption and replication independently from HO-1 induction [174]. However, the current literature regarding HO-1′s antiviral effect against SARS-CoV-2 remains unclear. Maestro et al. showed that hemin does not inhibit SARS-CoV-2 viral replication in vitro [175]. Kidney epithelial Vero-E6 and lung Calu3 cell lines were treated with hemin and results showed that, despite a strong activation of HO-1 in both cell lines, there was no effect on SARS-CoV-2 viral replication, measured by the amplification of the N viral gene by RT-qPCR [175]. However, a more recent report proposed hemin as a potential drug for treating COVID-19 via HO-1 induction [176]. Interestingly, authors observed a reduction in SARS-CoV-2 replication, both when pretreating and after SARS-CoV-2 infection treatment of Vero76 with this drug. Genetic induction or silencing of HO-1 in Vero76 cells demonstrated that the antiviral effect of hemin relies on this protein. Strikingly, this effect was mediated not only by Fe^2+^ and BV, but also by an HO-1 enzymatic independent mechanism. Further, they showed that hemin induced HO-1 boosted ISG15, OAS1 and MX1 protein expression in SARS-CoV-2 infected cells, highlighting the importance of stimulating the host cell’s IFN response against this virus [176]. Of note, there are reports from our laboratory showcasing that *MX1* gene expression is increased in COVID-19 patients. However, *MX1* expression is lower in elderly patients, where the disease has been shown to be more severe than in younger people. Additionally, through an in depth proteomics analysis, we described MX1 as a novel HO-1 interactor in prostate cancer (PCa) cell lines [177]. Moreover, genetic and pharmacological HO-1 induction in PCa cells triggered an increase in MX1 at mRNA and protein levels, and altered HO-1 cellular localization, showcasing a clear association between both proteins. Further, *MX1* silencing with a specific siRNA significantly decreased the expression of ERS genes (*HSPA5*, *DDIT3* and *XBP1*), demonstrating the role of MX1 in pro-death events [177].

In summary, the induction of the host infected cells antiviral response appears to be critical for COVID-19 treatment, which could be partly achieved by hemin mediated HO-1 induction, also preventing viral adsorption and replication by binding SARS-CoV-2 proteins. These antiviral effects are mediated by canonical and noncanonical HO-1’s functions. 

## 8. HO-1 Induction as a Strategy against COVID-19

There are mainly two different approaches to develop antiviral therapies: (1) therapies directed against viral factors; or (2) therapies targeting the host immune system. To date, the second strategy has received increasing attention due to the fact that targeting viral factors might cause viruses to mutate, increasing the rate of resistance to antiviral drugs [178]. In contrast to the viral genome, host cells’ DNA does not have a high mutational frequency. Therefore, overpowering viral infection by targeting host factors involved in the antiviral response is conceivably an effective strategy to counteract the severe consequences, while also fighting the infection [179].

During the last two years, several reports have focused on the understanding of the virus–host interaction underlying COVID-19 disease. The worrying situation of the SARS-CoV-2 pandemic and the threat of new variants, such as Omicron, which is spreading across the globe at an unprecedented rate, drive the interest of scientists to seek for new anti-SARS-CoV-2 strategies. Its enhanced transmissibility compared to the Delta variant could be explained in part by its increased rate of replication in human primary airway cultures, higher binding to ACE2, and ability to efficiently enter cells in a TMPRSS2-independent manner [180]. Fortunately, preliminary data of the Omicron variant suggest a lower virus load in both lower and upper respiratory tract, associated to less inflammatory processes in the lungs, using a mouse model of severe disease [181]. However, exceptionally high transmissibility could result in a great burden on healthcare systems across the globe. In this context, HO-1 emerges as a potential target to boost the host’s response to fight the infection and prevent severe COVID-19.

Certainly, HO-1 and its reaction products possess beneficial effects for the host during viral infections: it reduces inflammation and exerts antiviral actions. The most serious COVID-19 complications are: sepsis like inflammation, coagulopathy, and cardiovascular or respiratory complications. Furthermore, respiratory failure triggers hypoxia which, in combination with neuroinflammation, generates neurological complications [182]. When inflammation is not modulated, it turns into hyperinflammation and results in tissue damage or organ failure [183]. Enhancing HO-1 expression might help avoid the severe consequences of this disease. For example, it has been reported that HO-1 induction decreases inflammation, inhibits platelet aggregation, and increases fibrinolysis and phagocytosis, thus preventing tissue damage, thrombosis and sepsis [184]. Additionally, hemin is an activator of neuroglobin, a protein involved in oxygen transport and storage in neurons that increases oxygen’s intracellular partial pressure in neurons, and is crucial to protect neurons from hypoxic injury [185,186,187]. In addition, as mentioned above, HO-1 has a reported antiviral activity against multiple viruses. This effect depends on its classical activity involving its reaction subproducts (BR, BV, CO and Fe^2+^) and the activation of the IFN pathway; interestingly, its noncanonical activity is also involved in the antiviral effect of HO-1.

HO-1 expression is essentially regulated at the transcriptional level by NRF2. It has been reported that SARS-CoV-2 infection suppresses the NRF2 antioxidant gene expression pathway, and that NRF2 agonists limit viral replication and repress the proinflammatory response of SARS-CoV-2 [188]. This evidence highlights the relevance of the NRF2 signaling pathway on the antiviral response, suggesting that the activation of NRF2 might be a useful strategy against COVID-19 [189].

**Figure 4:**
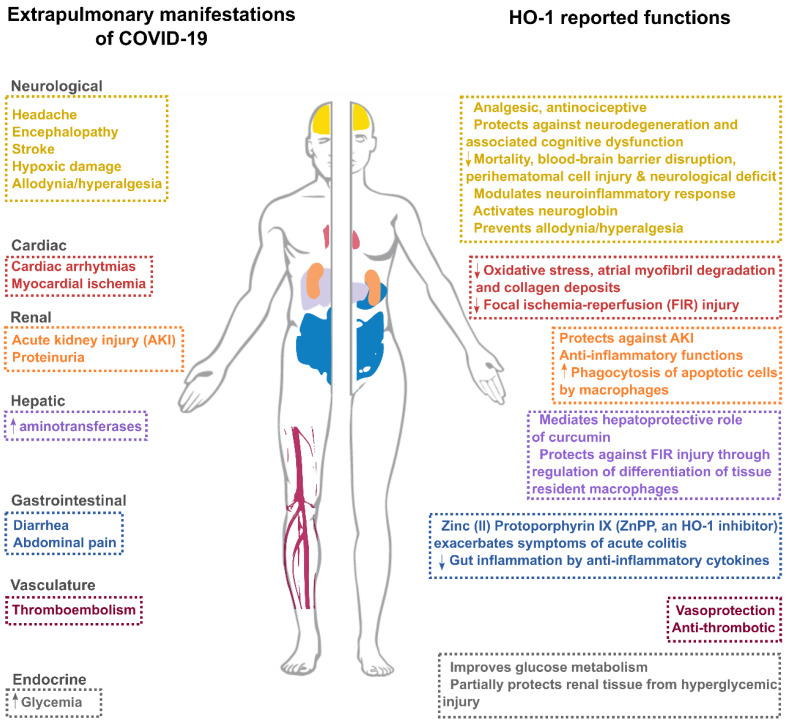
HO-1′s role in different sites that can be affected upon SARS-CoV-2 infection. Extra pulmonary manifestations of COVID-19 are grouped according to their site or body system. HO-1′s reported functions in different experimental conditions or diseases are grouped according to the model or system in which they are studied. The image of the human body has been adapted from Uhlén et al. (Human Protein Atlas, proteinatlas.org) [208].

As explained before, clinical complications associated with COVID-19 disease have been described in different organs, including vascular, cardiac, renal, hepatic, endocrine and neurological complications [190] (Figure 4). Interestingly, HO-1 has been reported to be associated with a reduction in tissue damage, mainly through its anti-inflammatory and antioxidative functions in different organs [4,97,147,191,192,193,194,195,196,197,198,199,200,201,202,203,204,205,206,207] (Figure 3). It would be interesting to address HO-1′s vasoprotective and antithrombotic effects for the prevention of thromboembolic events caused by SARS-CoV-2.

## 9. Closing Remarks

Drug repurposing is an attractive proposition, since it involves the use of derisked and previously approved compounds, with lower development costs and shorter development times [209]. Since the onset of the COVID-19 pandemic, we have witnessed a plethora of alternative drugs as potential therapeutic avenues to fight the disease. Thus, hemin, a previously FDA and EMA approved drug for acute intermittent porphyria treatment, rises as a promising drug candidate, inducing HO-1 plasma concentration in humans, and posing a host defense advantage to fight SARS-CoV-2. Further work on optimal drug concentrations, pharmacokinetics and pharmacodynamics should be performed in order to prove hemin’s effectiveness (either alone or in combination with other drugs) to halt infection.
